# Retinal Structures in Autism Spectrum Disorder: Results from a Case-Control Study

**DOI:** 10.1016/j.xops.2025.100842

**Published:** 2025-06-03

**Authors:** Fateme Montazeri, Yin Allison Liu, Parisa Emami-Naeini

**Affiliations:** 1 Tschannen Eye Institute, University of California, Davis, Sacramento, California; 2 Departments of Neurology and Neurosurgery, University of California, Davis, Sacramento, California

**Keywords:** Autism spectrum disorder, Autistic adults, Optical coherence tomography, Retinal structure

## Abstract

**Objective:**

To assess retinal structures in patients with autism spectrum disorder (ASD) and its correlation with cognitive impairments and brain volumes.

**Design:**

A retrospective case-control study.

**Subjects:**

Adults with ASD and matched neurotypical controls were identified from the UK Biobank (UKBB). The exclusion criteria included a history of neurodegenerative diseases, optic nerve pathology, retinal disorders, glaucoma surgery, high refractive error, or intraocular pressure outside the range of 6 to 21 mmHg.

**Methods:**

Using OCT images, 9 distinct retinal layers were segmented: the retinal nerve fiber layer (RNFL), ganglion cell layer, inner plexiform layer, inner nuclear layer (INL), combined outer plexiform layer and outer nuclear layer, photoreceptor inner segment, photoreceptor outer segment, retinal pigment epithelium, and choroidoscleral interface. Cognitive function was evaluated using 4 standardized tests: pairs matching, prospective memory, numerical or verbal reasoning, and reaction time. Additionally, brain imaging–derived phenotypes from the UKBB were included in the analysis. Generalized linear models were used to evaluate associations.

**Main Outcome Measures:**

Differences in retinal layer thickness between autistic individuals and controls, and the association with cognitive impairment and brain volumes.

**Results:**

We examined 240 eyes, including 80 from autistic participants and 160 from matched neurotypical controls. Autistic participants showed significantly higher thickness in the inner retina (adjusted mean differences: 5.71 μm, 95% confidence interval [2.49–8.93], *P* = 0.001), as well as RNFL (2.52 μm [0.97–4.06], *P* = 0.001), inner plexiform layer (1.18 [0.28–2.07], *P* = 0.010), and INL (0.93 [0.22–1.66], *P* = 0.010). No significant correlation was found between inner retinal thickness and cognitive impairment. However, brain magnetic resonance imaging data indicated associations between inner retinal thickness and volumes of the total brain, corpus collosum, hippocampus, and temporal gyrus.

**Conclusions:**

The inner retina may offer valuable insights into neurodevelopmental features in ASD, with observed associations with specific brain volumetric measurements. These findings could inform future research on ASD diagnostics and treatment.

**Financial Disclosure(s):**

Proprietary or commercial disclosure may be found in the Footnotes and Disclosures at the end of this article.

Autism spectrum disorder (ASD) is a heterogeneous neurodevelopmental disorder affecting >28 million individuals globally, with a growing incidence in developed countries.^1^ Diagnosing ASD can be complex, and there is evidence suggesting that individuals with milder forms of autism may receive a delayed diagnosis or may not be diagnosed at all.^2^ The lack of diagnosis can significantly affect the patient's functioning, increasing the risk of social deprivation, isolation, and various physical and mental health problems,^3^ which highlights the importance of effective diagnostic tools for timely and accurate diagnosis while limiting unnecessary referrals.^4^^,^^5^

Previous studies have established connections between the behavioral symptoms of ASD and biological variations in the brain, including differences in volume, structure, and connectivity across various regions.^6^ The retina—an extension of the CNS with structural and embryological similarities to the brain—can be noninvasively imaged in vivo and has recently emerged as a potential window into neurological conditions such as multiple sclerosis, Parkinson’s disease, Alzheimer’s disease, and schizophrenia.^7^ These findings highlight the potential of retinal imaging as an early diagnostic tool for neurological disorders. However, research on retinal structure in autistic individuals remains limited and, in some cases, controversial.^8^, ^9^, ^10^, ^11^ Additionally, the association between retinal anatomy and brain volume and structure is not well established.

In the present study, we used data from the UK Biobank (UKBB),^12^ a large population-based study, to investigate differences in retinal structure among participants on the autism spectrum compared with neurotypical individuals using OCT. Furthermore, we evaluated the association between cognitive function and brain volume measures with retinal layer thickness. The overarching goal of this study was to explore the diagnostic potential of retinal imaging in ASD, with the aim of identifying new biomarkers that could support diagnosis and treatment. This comprehensive approach may deepen our understanding of the neurobiological underpinnings of ASD and aid in the development of noninvasive adjunct diagnostic tools.

## Methods

In this retrospective case-control study, we obtained data from the UKBB (http://www.ukbiobank.ac.uk) under project application number 72417. The UKBB contains longitudinal data on over half a million participants aged 40 to 69 years recruited between 2006 and 2010. The UKBB recruited individuals registered with the National Health Service from 22 study centers across Scotland, England, and Wales.^13^ Participants completed comprehensive health questionnaires covering sociodemographic details, lifestyle factors, environmental exposures, medical history, family background, psychosocial factors, and cognitive function.^14^

A subset of 117 175 participants underwent eye examinations as part of the baseline evaluations between 2009 and 2010, with follow-up examinations conducted between 2012 and 2013.^15^ These examinations included assessments of visual acuity, noncycloplegic refraction (RC5000 auto refractometer, Tomey),^16^ corneal hysteresis, and intraocular pressure (IOP) (using the Ocular Response Analyzer, Reichert Corp) with adjustment for the biomechanical characteristics of the cornea using unique formulas to derive corneal-compensated IOP (IOPcc).^17^ Additional tests included color fundus photographs and macular OCT (Topcon 3D OCT 1000 Mk2, Topcon, Inc).^18^ The study followed the tenets of the Declaration of Helsinki and received approval from the Northwest Research Ethics Committee in the United Kingdom. All participants provided informed written consent. The present study's protocol was approved by the University of California Davis Institutional Review Board (IRB # 1791297-1). To protect participant confidentiality and minimize the risk of reidentification, categories with <10 individuals were suppressed.

### Cohort Description

We identified all individuals diagnosed with childhood autism, atypical autism, or Asperger syndrome based on the International Classification of Diseases 10th revision codes F84.0, F84.1, and F84.5, as well as those diagnosed with autism, Asperger, or autistic spectrum disorder by a mental health professional through online follow-up (UKBB 20544). From this group, we generated our ASD cohort by including participants who had retinal OCT images available in the UKBB. An age- and sex-matched control cohort was created, consisting of participants with no prior diagnosis of autism. For both ASD and control groups, we excluded participants who had withdrawn consent or had diagnosis of optic nerve or visual pathway pathology (International Classification of Diseases-10: H46–48), history of retinal pathology (e.g., retinal detachments and breaks [H33], retinal vascular occlusions [H34], other retinal disorders [H35], retinal disorders in diseases classified elsewhere [H36]), or neurodegenerative diseases (e.g., Parkinson’s disease, Alzheimer’s disease, and demyelinating diseases of the CNS). We also excluded individuals who self-reported a history of glaucoma, macular degeneration, or retinal pathologies, as well as those with a history of vitreoretinal or glaucoma surgery. Participants with high refractive error (spherical equivalent refraction of less than −6 diopters or greater than +6 diopters) and those with IOPcc measurements <6 mmHg or >21 mmHg were also excluded.

### OCT

Spectral-domain OCT scans (Topcon) were obtained in a dark room without pupillary dilation, using a raster scan protocol of 128 B-scans with 512 horizontal A-scans per B-scan, centered on the fovea and covering a 6 × 6 mm field. Three-dimensional macular volume scans were saved as .fda files without prior thickness analysis.^18^ We used the Topcon Advanced Boundary Segmentation algorithm^19^ for automatic segmentation of the retina into the following boundaries and layers: the internal limiting membrane, retinal nerve fiber layer (RNFL), ganglion cell layer, inner plexiform layer, inner nuclear layer (INL), outer plexiform layer plus outer nuclear layer, photoreceptor inner segment (IS), photoreceptor outer segment, retinal pigment epithelium, and choroidal-scleral interface. Images with software-generated quality scores <40 were excluded from the analysis. Retinal layer thickness was determined by calculating the distance between the boundaries of interest and averaging these values across all scans.

### Cognitive Performance

Cognitive function was assessed using unsupervised automated touch screen questionnaires based on 13 different tests, which included reaction time, numeric memory, intelligence or reasoning, trail making, matrix pattern completion, tower rearranging, picture vocabulary, symbol digit substitution, paired associate learning, prospective memory, pairs matching, lights pattern memory, and word production.^20^ These tests were designed for automated administration within the UKBB framework. Impaired cognition was defined using criteria outlined by Ko et al,^21^ which identified individuals as cognitively impaired if they met ≥1 of the following criteria: (1) >2 incorrect matches in the pairs-matching test, (2) an incorrect response on the initial attempt for the memory test, (3) a score of <3 in numerical and verbal reasoning tests, or (4) a reaction time >770 ms.

### Brain Magnetic Resonance Imaging

Brain magnetic resonance imaging (MRI) data were acquired using 3T Siemens Skyra scanners (software VD13) with the standard Siemens 32-channel receive head coil.^22^ We used the global and regional MRI derived phenotypes provided by the UKBB imaging team, which were based on T1-weighted imaging. These measurements included total brain volume (the combined gray and white matter volume, excluding cerebrospinal fluid; data field: 25009, gray matter volume [25005], white matter volume [25007], peripheral cortical gray matter volume [25001], and ventricular cerebrospinal fluid volume [25003]). Subcortical volumes were measured using Freesurfer Automated Segmentation tool, which included the cerebellum white matter (26556, 26587), cerebellum cortex (26557, 26588), whole brain stem (26526), and corpus callosum (26531–26535). Additionally, subcortical nuclei volumes were measured using FIRST, which encompassed the accumbens (25023, 25024), amygdala (25021, 25022), caudate (25013, 25014), hippocampus (25019, 25020), pallidum (25017, 25018), putamen (25015, 25016), and thalamus (25011, 25012). Gray matter cortical segmentations were derived from the Harvard–Oxford Atlas using FMRIB's Automated Segmentation Toll.^23^

### Statistical Analysis

We used chi-square test and Student *t* test to compare demographics and baseline characteristics between the ASD and control groups. Retinal layer thicknesses were compared using a robust generalized linear model. If both eyes of a participant were eligible for inclusion, we averaged the measurements of the 2 eyes. The model was adjusted for covariates such as age, sex, smoking status, visual acuity, refraction, and IOPcc. We checked for multicollinearity using the covariance matrix and addressed missing data in refraction and IOP by imputing the mean value. After standardizing the data, we used a generalized linear model with a binomial family distribution to assess the association between retinal thickness and cognitive impairment. Additionally, the association between brain volumes and retinal thickness was examined using generalized linear models. Before analysis, all MRI measures were normalized for head size by scaling individual brain imaging-derived phenotypes using volumetric scaling from T1 head images to standard space. Brain and retina measurements were then standardized by subtracting the mean value from the observation and dividing by the standard deviation. Multiple testing correction was applied using the Benjamini–Hochberg method.^24^ Statistical analysis was conducted using Python (Python Software Foundation, https://www.python.org/). All analyses were 2-sided, with a *P* value <0.05 considered statistically significant.

## Results

### Characteristics of Cohorts

We identified a total of 421 participants diagnosed with autism, of whom OCT images were available for 79 participants (157 eyes). After excluding 71 eyes due to poor image quality and an additional 6 eyes based on other predefined exclusion criteria, a total of 240 eyes (80 eyes from 49 autistic individuals and 160 eyes from 86 matched controls) were included in the analysis. There was no statistically significant difference between the autistic and nonautistic groups regarding age, sex distribution, ethnic background, smoking history, visual acuity, refractive error, or IOPcc (Table 1).

**Table 1:** Participants Baseline Characteristics∗

	Case (n = 49 Individuals per 80 Eyes)	Control (n = 86 Individuals per 160 Eyes)	*P* Value
Age, yr	49.8 ± 7.3	50.6 ± 7.6	0.59
Sex, female	15 (30.6)	21 (24.4)	0.56
Ethnic background			
White	48 (98)	83 (96.5)	1.00
Black or British Black	<10 (<3)	<10 (<4)	
Asian or British Asian	<10 (<3)	<10 (<4)	
Chinese	<10 (<3)	<10 (<4)	
Mixed	<10 (<3)	<10 (<4)	
Other	<10 (<3)	<10 (<4)	
Smoking history			
Current or past smoking	25 (51)	30 (34.8)	0.09
Never smoked	24 (49)	56 (65.1)	
Ocular examination			
Visual acuity (LogMAR)	−0.01 ± 0.2	−0.04 ± 0.1	0.31
Refractive error (D)	−0.10 ± 1.5	0.03 ± 1.3	0.46
IOP (mmHg)	14.42 ± 2.8	14.82 ± 2.9	0.30
Cognitive function tests			
Fluid intelligence score	6.79 ± 2.3	6.95 ± 2.1	0.69
Reaction time test (ms)	538.18 ± 161.3	531.12 ± 102.9	0.75
Prospective memory failed	10 (20.4)	11 (12.8)	0.35
Pairs matching failed	0	<10 (<9)	0.29

D = diopter; IOP = intraocular pressure; LogMAR = logarithm of the minimum angle of resolution; mmHg = millimeter of mercury; ms = millisecond.

∗ The table is formatted by mean ± standard deviation or number (%). Where appropriate, *P* values are obtained by *t* test, chi-square test, and Fisher exact test.

### Retinal Layer Thickness

Autistic individuals had a significantly higher RNFL thickness compared with the control group (adjusted mean difference [aMD]: 2.52 μm , 95% confidence interval [CI] [0.97–4.06], *P* = 0.001). Increased thickness was also observed in the inner plexiform layer (aMD: 1.18, 95% CI [0.28–2.07], *P* = 0.010) and INL (aMD: 0.93, 95% CI [0.22–1.66], *P* = 0.010). Additionally, a significantly thicker inner retina (between internal limiting membrane and INL / outer plexiform layer) was found among autistic participants compared with controls (aMD: 5.71, 95% CI [2.49–8.93], *P* = 0.001). Although an increase in the ganglionic cell layer thickness was observed in the autistic group (aMD 1.07, 95% CI [0.16–1.97], *P* = 0.021), this did not reach statistical significance after adjustment for multiple testing. There was no statistically significant difference in the thickness of the outer retinal layers including outer plexiform layer + outer nuclear layer, IS, outer segment, and retinal pigment epithelium, between autistic and nonautistic participants (Table 2).

**Table 2:** Comparison of Macular Thickness Measurements (μm)

Retinal Layer	Case Mean ± SD	Control Mean ± SD	Adjusted Mean Difference∗	95% Confidence Interval	*P* Value
Retinal nerve fiber	42.01 ± 4.41	40.01 ± 5.99	2.52	0.97–4.06	0.001
Ganglion cell	34.11 ± 2.09	33.37 ± 3.67	1.07	0.16–1.97	0.021
Inner plexiform	29.93 ± 2.45	28.93 ± 3.31	1.18	0.28–2.07	0.010
Inner nuclear	31.44 ± 2.02	30.69 ± 2.91	0.93	0.22–1.66	0.010
Outer plexiform + nuclear	75.70 ± 5.23	77.6 ± 7.38	−1.56	−3.59 to 0.47	0.133
Photoreceptor (inner segment)	23.03 ± 1.15	23.41 ± 1.38	−0.36	−0.78 to 0.05	0.084
Photoreceptor (outer segment)	39.79 ± 2.71	39.73 ± 3.80	0.36	−0.67 to 1.39	0.493
Retinal pigment epithelium	23.14 ± 2.02	23.80 ± 6.10	−0.72	−1.76 to 0.32	0.175
Choroidal-scleral interface	220.91 ± 40.11	216.61 ± 50.24	5.67	−7.23 to 18.58	0.389
Total inner retina	137.51 ± 7.15	133.03 ± 14.11	5.71	2.49–8.93	0.001
Total outer retina	138.52 ± 7.08	140.76 ± 9.76	−1.57	−4.19 to 1.05	0.241
Total retina	299.18 ± 10.47	297.6 ± 17.56	3.42	−0.95 to 7.79	0.126

SD = standard deviation.

∗ Adjusted for age, sex, smoking status, logarithm of the minimum angle of resolution, refraction, and intraocular pressure.

### Cognitive Function and Retinal Thickness

**Figure 1:**
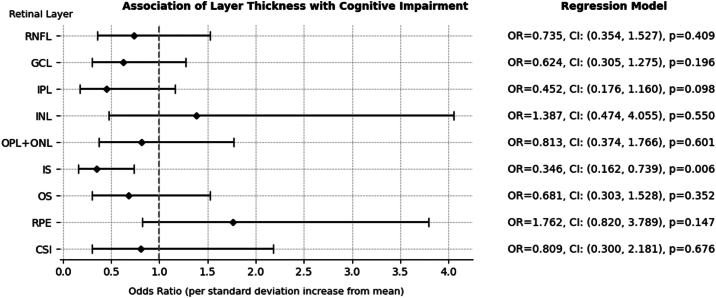
Retinal layer thickness and cognitive impairment in autistic participants. The odds ratio of cognitive impairment with a standard deviation increase of thickness from the average in each layer is presented. CI = confidence interval; CSI = choroidal-scleral interface; GCL = ganglion cell layer; INL = inner nuclear layer; IPL = inner plexiform layer; IS = inner segment; ONL = outer nuclear layer; OPL = outer plexiform layer; OR = odds ratio; OS = photoreceptor outer segment; RNFL = retinal nerve fiber layer; RPE = retinal pigment epithelium.

We used logistic regression models to evaluate the association between retinal thickness and cognitive function in autism. Among 49 autistic participants, 12 (24.4%, 22 eyes) were identified as cognitively impaired. Our analysis showed that thinner IS layer was associated with variations in cognitive performance (adjusted odds ratio: 0.34, 95% CI [0.16–0.74], *P* = 0.006). No clear association was observed between the thickness of other retinal layers and the cognitive abilities of autistic individuals (Fig 1).

### Brain MRI and Retinal Thickness

**Figure 2:**
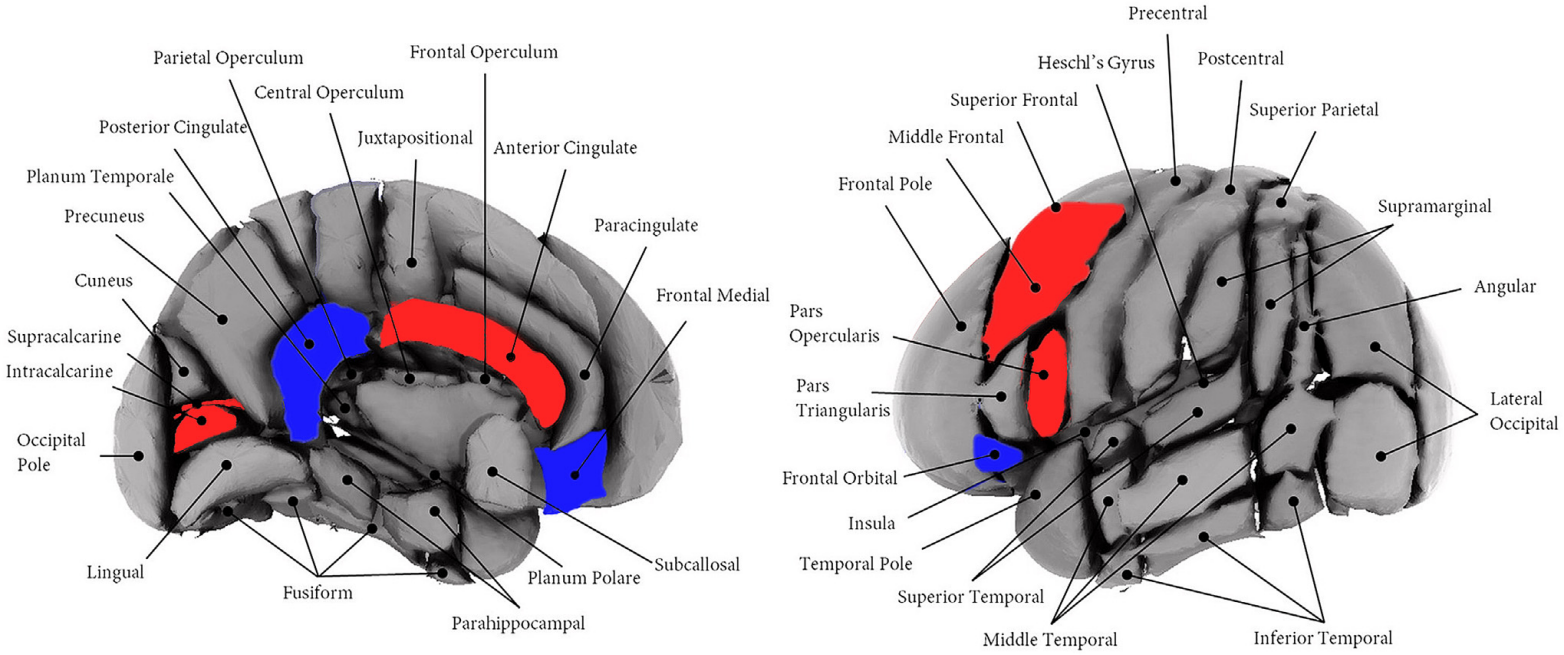
Association of retinal nerve fiber layer thickness and cortical regions of interest, according to the Harvard–Oxford Atlas. Positive correlations are shown in red and negative correlations are shown in blue. Figure adapted from Cox et al,^25^ with color modifications.

Brain MRI measurements were available for 7 autistic participants (14.3%, 11 eyes) and 35 controls (40.7%, 64 eyes). In the autism group, we found a positive correlation between inner retinal thickness and total brain volume (β = 0.47, 95% CI [0.22–0.73], *P* < 0.001) as well as with the posterior part of the corpus callosum (β = 0.67, 95% CI [0.32–1.03], *P* < 0.001). Conversely, the volume of the hippocampus and the gray matter in the anterior division of the inferior temporal gyrus showed negative correlations with inner retinal thickness (β = −0.64, 95% CI [−0.99 to −0.29], *P* < 0.001; β = −0.67, 95% CI [−0.99 to −0.34], *P* < 0.001). Additionally, RNFL thickness was positively correlated with ventricular cerebrospinal fluid volume (β = 0.70, 95% CI [0.23–1.16], *P* = 0.003) and various gray matter volumes, including total gray matter (β = 0.64, 95% CI [0.24–1.05], *P* = 0.002), the anterior division of the cingulate gyrus (β = 0.79, 95% CI [0.51–1.08], *P* < 0.001), the intracalcarine cortex (β = 0.76, 95% CI [0.23–1.30], *P* = 0.005), the supracalcarine cortex (β = 0.69, 95% CI [0.33–1.05], *P* < 0.001), the pars opercularis of the inferior frontal gyrus (β = 0.60, 95% CI [0.41–0.79], *P* < 0.001), and the middle frontal gyrus (β = 0.75, 95% CI [0.35–1.16], *P* < 0.001). Conversely, negative correlations with RNFL thickness were found for the volume of the cerebellar cortex (β = −0.33, 95% CI [−0.55 to −0.12], *P* = 0.002), the mid-posterior corpus callosum (β = −0.83, 95% CI [−1.04 to −0.62], *P* < 0.001), the gray matter of the frontal medial cortex (β = −0.72, 95% CI [−1.19 to −0.25], *P* = 0.003), and the frontal orbital cortex (β = −0.67, 95% CI [−0.92 to −0.42], *P* < 0.001) (Fig 2, Table S3, available at www.ophthalmologyscience.org). We found no statistically significant differences in the volumes of these brain regions between cases and controls. Furthermore, no correlation was observed between the thickness of retinal layers and the volume of brain regions of interest among the neurotypical controls.

## Discussion

In this study, we investigated the potential of retinal thickness measurements as accessible, noninvasive indicators to aid in diagnosing autism. Given the current diagnostic challenges—including long waiting periods and varied diagnostic practices—alternative assessment approaches are essential.^26^ We observed a significant increase in inner retinal layer thickness in autistic adults, specifically the RNFL, inner plexiform layer, and INL. Notably, increased inner retinal thickness correlated positively with total brain volume and the posterior corpus callosum, while showing negative correlations with the hippocampus and the inferior temporal gyrus. Additionally, RNFL thickness showed positive correlations with several brain regions, including total gray matter, anterior cingulate gyrus, intracalcarine cortex, supracalcarine cortex, inferior frontal gyrus, and middle frontal gyrus. In contrast, it was negatively associated with the cerebellar cortex, mid-posterior corpus callosum, the frontal orbital cortex, and the medial frontal cortex in autistic individuals. Interestingly, cognitive function did not correlate with inner retinal thickness, although a decrease in the photoreceptor IS thickness was linked to a higher likelihood of cognitive impairment in individuals on the autism spectrum.

Previous studies on retinal structure in autistic populations have included small, predominantly younger cohorts and reported mixed results. Gialloreti et al^8^ examined peripapillary RNFL (pRNFL) thickness in young adults with ASD and found thinning in the nasal quadrant compared with controls, although global pRNFL thickness did not differ significantly. When patients were stratified into high-functioning autism and Asperger syndrome subgroups, only the high-functioning ASD group demonstrated significantly thinner global pRNFL. In another study of children with ASD, reduced global pRNFL thickness was observed, while macular thickness remained comparable to that of controls.^27^ In contrast, Friedel et al^11^ reported significantly reduced macular and outer nuclear layer thickness in adults with ASD compared with neurotypical controls. Conversely, Garcia-Medina et al^9^ found increased macular thickness—including both total and inner retinal layers—and thicker pRNFL in children with ASD. Follow-up research by the same group confirmed a trend toward overall retinal thickening in this population.^10^ These conflicting results likely reflect differences in study populations, including age, cognitive profiles, and ASD subtypes, as well as methodological variation, highlighting the need for further investigation to clarify the retinal phenotype in ASD.

Our findings of increased inner retinal thickness in autistic adults contribute to this evolving body of literature. Some discrepancies with previous studies may be attributed to the heterogenicity of ASD, differences in study populations,^9^ retinal imaging techniques,^10^ and statistical methodologies.^8^ While earlier research primarily focused on pediatric^9^^,^^10^^,^^27^ and young adult cohorts,^8^^,^^11^ our study uniquely examines older adults and includes a larger sample size. Additionally, by averaging measurements from both eyes, we minimized intereye correlation bias—a potential contributor to type I error.^28^ Differences in OCT devices used^29^ and retinal segmentation algorithms^30^ may also contribute to measurement variability, particularly in autistic individuals who may have difficulty maintaining optimal fixation during imaging.

The observed retinal thickening in autism may result from neuroinflammatory changes, such as microglial activation,^31^ vascular anomalies,^10^ or may reflect atypical neurodevelopmental processes in the brain.^32^ Some studies have highlighted accelerated brain growth during the first 2 years of life in ASD, which may normalize or persist into adulthood.^33^^,^^34^ While we did not observe significant differences in overall brain volume between autistic and nonautistic individuals, our results showed associations between increased inner retinal thickness and brain volumes in autistic adults, suggesting a potential link between retinal structure and localized neuroanatomical alterations in the brain.^32^

Autism is often marked by distinct visual processing characteristics, with proficient or enhanced static spatial abilities and challenges in more complex dynamic tasks^35^ that require cross-region communication beyond the primary visual cortex.^36^ The inferior temporal gyrus, responsible for higher-order visual perception, such as recognizing patterns based on visual categories,^37^ shows structural variations in ASD^38^ and demonstrated a negative association with inner retinal thickness in our study. Similarly, we observed a negative correlation between inner retinal thickness and the volume of the hippocampus, which plays a key role in memory, language skills, and social-emotional communication.^39^ Volumetric abnormalities in the hippocampus have been linked to core features of ASD, including differences in language development.^39^

The RNFL, comprised primarily of ganglion cell axons that converge into the optic nerve and connect to the CNS, frequently shows alterations in neurological conditions.^7^ Our findings of correlations between RNFL thickness and volumes of cortical areas—such as the occipital and frontal lobes—suggest a complex interplay between retinal and cortical structures in ASD. Atypical development of the visual cortex during the early stages of ASD may disrupt the transmission of visual information from retinal ganglion cells to the cortex.^40^ In line with prior research indicating increased gray matter volume in visual processing areas in ASD,^41^ our results showed a positive association between RNFL thickness and volumes of the intracalcarine and supracalcarine cortex—regions of the occipital lobe responsible for orientation, spatial, and color perception.^42^ Conversely, negative correlations with the orbitofrontal and medial frontal cortex are consistent with observed differences in cortical activity across these regions in individuals with ASD.^43^

Our study has several limitations. First, we focused only on macular measurements due to limited data available on the peripapillary area in our dataset. Second, the predominance of autistic participants who do not require significant support, along with an ethnically homogenous sample, may limit the generalizability of our findings to the broader autism spectrum. Additionally, the nonspecific cognitive assessments used in the UKBB may not have fully captured the differences across ASD subpopulations. The relatively small subset of participants with available MRI data introduces potential bias. Structural brain analysis is inherently complex, given region-specific, nonlinear growth trajectories and substantial interindividual variability, even within normative development. We evaluated brain volumes exclusively through T1-weighted imaging, which may not reflect other neurodevelopmental characteristics, such as brain function or microstructural integrity. Future studies with larger sample sizes are needed to address these limitations. Moreover, because our cohort consisted only of adults, this study does not assess whether retinal imaging can support ASD diagnosis in early childhood. Longitudinal studies in younger populations are needed to evaluate the age-dependent relevance of these findings.

In conclusion, our findings suggest that OCT may serve as a valuable addition to existing diagnostic tools for ASD by providing insights into the associations between retinal structure and brain morphology. This study represents preliminary evidence, highlighting a potential avenue for future research aimed at developing noninvasive adjunct assessment tools. The observed complex correlations between retinal layers and cortical regions involved in visual processing underscore the need for further investigation to elucidate the mechanisms underlying these associations, particularly in ASD subgroups with shared clinical and neuroanatomical characteristics.
