# Supplementary material for: Retinal Structures in Autism Spectrum Disorder: Results from a Case-Control Study
Source: Ophthalmol Sci. 2025 Jun 3;5(6):100842. doi: 10.1016/j.xops.2025.100842 (PMC12395168; doi:10.1016/j.xops.2025.100842)
Supplement: Table S3 [file mmc1.pdf]

Table S3- Significant Associations Between Brain Volumes and Retinal Thicknesses in Autistic Participants [a]

Brain Region of Interest	Volume, mean (SD), mm^3		Coefficient	Confidence Interval		P value
	Case	Control	Inner Retina			
Hippocampus	4906.26 (721)	4995.1 (486.2)	-0.6438	-0.993	-0.295	<0.001
Grey matter in Inferior Temporal Gyrus, Anterior division	2076.05 (240.8)	1978.85 (307.8)	-0.6709	-0.994	-0.348	<0.001
Corpus callosum, Posterior	1381.66 (79.8)	1359.94 (160.9)	0.6781	0.327	1.029	<0.001
Total brain	1538738.75 (62249.8)	1521287.71 (75176.5)	0.4778	0.221	0.735	<0.001
			RNFL			
Grey matter in Middle Frontal Gyrus	13193.16 (1466.9)	12325.12 (1302.4)	0.7583	0.357	1.159	<0.001
Grey matter in Inferior Frontal Gyrus, Pars opercularis	3245.39 (640.3)	3436.43 (490.6)	0.6031	0.411	0.795	<0.001
Grey matter in Frontal Medial Cortex	2347.49 (216.8)	2500.88 (374.1)	-0.7201	-1.189	-0.251	0.003
Grey matter in Frontal Orbital Cortex	8443.93 (349.3)	8335.18 (811.2)	-0.6746	-0.928	-0.421	<0.001
Grey matter in Supracalcarine Cortex	815.2 (146.7)	799.17 (149.4)	0.6947	0.332	1.057	<0.001
Grey matter in Intracalcarine Cortex	3439.48 (746.8)	3524.46 (669.3)	0.7662	0.232	1.3	0.005
Grey matter in Cingulate Gyrus, Anterior division	6964.38 (1183.3)	7009.22 (1000.3)	0.7995	0.512	1.087	<0.001
Cerebellum Cortex	71980.35 (3935.6)	72431.22 (6275.4)	-0.338	-0.556	-0.12	0.002
Corpus Callosum, Mid Posterior	674.47 (93.1)	702.22 (174.7)	-0.8329	-1.041	-0.625	<0.001
Ventricular cerebrospinal fluid	43319.28 (17357.1)	41233.43 (20603.4)	0.7012	0.236	1.167	0.003
Total Grey matter	812621.62 (19880.3)	801561.2 (46057.4)	0.6449	0.242	1.048	0.002

[a] Coefficients, confidence intervals, and p-values are generated by generalized linear model.
